# Intervención para fomentar el cumplimiento normativo en educación alimentaria y nutricional en escuelas públicas de educación básica en la región central de México

**DOI:** 10.18294/sc.2025.5412

**Published:** 2025-08-14

**Authors:** José Cutberto Hernández Ramírez

**Affiliations:** 1 Doctor en Ciencias de la Salud. Investigador posdoctoral, Secretaría de Ciencia, Humanidades, Tecnología e Innovación, Ciudad de México, México. nutramedia76@outlook.es Secretaría de Ciencia, Humanidades, Tecnología e Innovación Secretaría de Ciencia, Humanidades, Tecnología e Innovación Ciudad de México Mexico nutramedia76@outlook.es

**Keywords:** Seguridad Alimentaria, Acceso a Alimentos Saludables, Organización Comunitaria, Participación de la Comunidad, Food Security, Access to Healthy Foods, Communitarian Organization, Community Participation

## Abstract

Las intervenciones de educación alimentaria y nutricional en escuelas de educación básica mexicanas se han centrado en transmitir información y en las infancias, pero obvian determinantes del aprendizaje de hábitos alimentarios, como el cumplimiento o la transgresión de normas oficiales sobre la oferta alimentaria en la escuela. Este artículo describe los resultados de una intervención educativa para fomentar el cumplimiento normativo favorable para la educación alimentaria y nutricional en cuatro escuelas públicas de educación básica en la región central de México, dirigida a profesorado y cocineras que construyen cotidianamente el ambiente alimentario escolar. Se realizó un estudio de caso comparativo de 2021 a 2023, mediante una serie de talleres temáticos con enfoque constructivista, adaptados a un diagnóstico previamente reportado y dirigidos a un total de 38 docentes, 8 cocineras y 105 asistentes eventuales (familiares del alumnado). La incidencia de los talleres se evaluó mediante un análisis ex-post de debilidades, amenazas, fortalezas y oportunidades, y con pruebas de hipótesis sobre los cambios en los menús escolares ofertados cotidianamente. La asistencia constante de las mismas personas a las sesiones de los talleres contribuyó a cambios significativos en la calidad nutricional de los menús ofertados, y las estrategias generadas en los talleres por el profesorado y las cocineras para intervenir cotidianamente en su entorno fueron las más eficaces. Los talleres con cambio constante de asistentes abarcaron más miembros de la comunidad, pero impactaron menos en el cumplimiento normativo favorable para la educación alimentaria y nutricional.

## Abstract (en)

Food and nutrition education interventions in Mexican basic education schools have focused on conveying information and targeting children, but they overlook determinants of learning eating habits, such as compliance with or violation of official regulations on food offerings in schools. This article describes the results of an educational intervention aimed at promoting regulatory compliance favorable to food and nutrition education in four public basic education schools in the central region of Mexico, directed at teachers and cooks who shape the school food environment on a daily basis. A comparative case study was conducted from 2021 to 2023 through a series of thematic workshops with a constructivist approach, adapted to a previously reported diagnosis, and addressed to a total of 38 teachers, 8 cooks, and 105 occasional attendees (students’ family members). The impact of the workshops was evaluated through an ex-post analysis of weaknesses, threats, strengths, and opportunities, as well as hypothesis testing on changes in the daily school menus offered. The consistent attendance of the same participants in the workshop sessions contributed to significant improvements in the nutritional quality of the menus offered, and the strategies generated in the workshops by teachers and cooks to intervene daily in their environment proved to be the most effective. Workshops with constantly changing attendees involved more members of the community but had less impact on regulatory compliance favorable to food and nutrition education.

## Introducción

La educación alimentaria y nutricional (EAN) es un conjunto de estrategias pedagógicas implementadas a niveles individual, grupal, institucional y nacional para contribuir en la mejora de las prácticas alimentarias^1^. Promueve entornos saludables y genera capacidades para que la gente consuma alimentos saludables y asequibles, identifique opciones alimentarias adecuadas y desfavorables e instruya a otras personas. No equivale a mejores conocimientos en nutrición ni a difundir información sobre alimentos y sus nutrientes, más bien, busca el aprendizaje de actitudes y comportamientos alimentarios^1^.

Ciertamente, las actitudes se aprehenden por la asimilación de aspectos en las relaciones interpersonales cotidianas, lo que hace imposible enseñarlas tan solo mediante las palabras^2^. Por ende, la coherencia entre el discurso y la práctica de la persona educadora conformaría la praxis educativa requerida para propiciar el aprendizaje de actitudes^3^, las cuales son posturas orientadas tanto por el conocimiento como por la afectividad^4^. 

Así, la educación alimentaria y nutricional genera prácticas alimentarias orientadas por conocimientos técnicos, pero también por pautas culturales sustentadas en los sentimientos involucrados en el cuidado personal y de los otros^2^, en la confianza en las propias acciones y las de los demás, en el respeto, en la empatía y en la colaboración para una coexistencia solidaria y comprometida en construir ambientes saludables^5^.

Con todo, las intervenciones de educación alimentaria y nutricional en México se han centrado en objetivos cognitivos. Dirigidas mayormente a las infancias en contextos escolares, siguen una secuencia de evaluación nutricional inicial, intervención (basada mayormente en transmisión de mensajes verbales reforzados por material impreso) y evaluación final, paralelas al currículo^6^^,^^7^^,^^8^^,^^9^^,^^10^.

Tales intervenciones desaprovechan el valor didáctico de la ingesta de alimentos en la escuela, la cocina demostrativa y/o participativa, y la integración sensorial^11^ mediante la comida. En cambio, son proclives a centrarse en una pedagogía mecanicista implícita al esperar cambios conductuales automáticos derivados de acumular información tal como se ha evidenciado que ocurre frecuentemente en Latinoamérica^12^.

Aunque desde el año 2010 el gobierno mexicano emitió directrices para suministrar alimentos y bebidas en las escuelas de educación básica^13^, en estas aún se ofertan alimentos con alta densidad energética y escaso valor nutricional impartiendo así un currículo oculto discordante con la educación alimentaria y nutricional^14^^,^^15^^,^^16^. Tal incumplimiento normativo revela insuficiente *compliance* o cumplimiento normativo (procedimientos y estrategias de las propias organizaciones para asegurar el cumplimiento de normas jurídicas) lo cual indicaría limitada responsabilidad social organizacional, entendida como el conjunto de valores morales expresados en prácticas favorables para la sostenibilidad de la sociedad^17^.

Así, dichos lineamientos y campañas del gobierno mexicano sustentadas mayormente en alfabetización nutricional y comunicación educativa^18^, orientadas al cumplimiento de normas oficiales, han generado insuficiente cumplimiento normativo en las escuelas de educación básica^16^. Esta discordancia entre deber ser (normas oficiales en materia de oferta de alimentos en las escuelas) y ser (oferta de alimentos discordante con las normas oficiales), podría deberse a la existencia de fenómenos de pluralismo normativo^19^, es decir, coexistencia de sistemas normativos alternos -y no escritos- en los espacios escolares. Asimismo, tal discordancia de mensajes contradictorios podría devenir en un efecto cacofónico en la construcción del saber-hacer alimentario^20^ en el ámbito escolar y derivar en confusión^21^ para la toma de decisiones alimentarias especialmente en el consumidor.

En suma, si bien la educación alimentaria y nutricional idealmente se centraría en lo cognitivo (conocimientos), afectivo (actitudes) y psicomotor (habilidades) para lograr la adopción voluntaria de prácticas alimentarias saludables, en los contextos mexicanos de educación básica reportados se ha centrado principalmente en aspectos cognitivos y solo en los estudiantes, lo cual se contradice con el currículo oculto implícito en la oferta de alimentos nutricionalmente inadecuados en los planteles escolares. Además, esta consistente transgresión de las disposiciones jurídicas vigentes, en materia de oferta de alimentos en las escuelas, sugiere la existencia de fenómenos de pluralismo normativo. Todo lo anterior resulta en un impacto limitado para construir entornos alimentarios escolares saludables.

Así, esta investigación partió de un diagnóstico previamente reportado sobre la red de significados que orienta la oferta de alimentos en los planteles^5^^,^^22^^,^^23^, el cual sirvió como base para diseñar e implementar la intervención educativa aquí reportada. De ahí, el objetivo del artículo es describir los resultados de una intervención educativa para fomentar el cumplimiento normativo favorable para la educación alimentaria y nutricional en cuatro escuelas públicas de educación básica en la región central de México, dirigida a profesorado y cocineras que colaboran directamente en la construcción cotidiana del ambiente alimentario escolar. 

## Materiales y métodos

Se realizó un estudio de caso comparativo en cuatro escuelas de educación básica del centro de México. Es oportuno detallar que, en términos generales, los estudios de caso comparativos abarcan dos o más casos y generan conocimiento sobre cómo y por qué determinadas intervenciones funcionan o no, según las características específicas de cada contexto comparado y orientan respecto a cómo adaptar las intervenciones en función del contexto^24^. Asimismo, son longitudinales y se centran en la comparación dentro y entre contextos, mediante el uso mixto de métodos cualitativos y cuantitativos^24^. Además, los estudios de caso comparativos son una alternativa viable cuando no es posible realizar diseños experimentales^24^. 

La selección de las cuatro escuelas participantes fue por conveniencia conforme a los siguientes criterios de inclusión: 1) ser públicas, 2) impartir servicios educativos a nivel preescolar y/o escolar, 3) haber desarrollado previamente y tener en curso estrategias para mejorar el estado de nutrición del alumnado, 4) contar con el servicio de restauración alimentaria, y 5) tener interés en participar en la investigación. 

Es oportuno comentar que, en el contexto de las escuelas públicas mexicanas de educación básica, con base en Araluce^25^, los servicios de restauración alimentaria pueden definirse como establecimientos que elaboran comida para su consumo fuera del hogar, orientados al sector social en el cual prevalece el servicio colectivo sobre la elección particular y que son parte de las prestaciones que ofrecen las escuelas. En México, estos servicios de restauración alimentaria son instaurados con recursos del gobierno federal y principalmente sirven para operar -con apoyo de los gobiernos estatal y municipal- el programa alimentario de desayunos escolares calientes, dirigido a infancias que asisten a escuelas públicas del sistema educativo nacional ubicadas en zonas con niveles importantes de pobreza y vulnerabilidad^26^. 

En este sentido, la pobreza se define como la condición en la que alguien tiene un ingreso insuficiente para adquirir bienes alimentarios y no alimentarios básicos además de presentar una o más carencias sociales, definidas respectivamente como falta de: acceso a servicios de salud, acceso a la seguridad social, calidad y espacios de la vivienda, servicios básicos en la vivienda, acceso a la alimentación, y rezago educativo^27^. Hay pobreza extrema cuando confluyen tres o más carencias sociales más un ingreso insuficiente para adquirir bienes alimentarios básicos^27^. En cambio, la vulnerabilidad es la condición en la que alguien presenta alguna de las siguientes dos características, pero no ambas: 1) una o más carencias sociales, o 2) ingreso igual o menor al requerido para adquirir bienes alimentarios y no alimentarios básicos^27^. 

Así, la caracterización general de las escuelas y de su contexto, en términos sociodemográficos, se realizó con base en información ofrecida por la dirección de cada plantel y en datos del Censo de Población y Vivienda 2020, disponibles en la web oficial gubernamental DataMEXICO^28^.

Por otro lado, en el diagnóstico previo a la intervención, cuyos hallazgos han sido publicados en tres reportes^5^^,^^22^^,^^23^, el objetivo fue identificar la red de significados (normas y reglas técnicas) que orienta la oferta de alimentos en la escuela, así como la estructura y la composición del menú cíclico ofertado en el servicio de restauración alimentaria. Tales hallazgos se conformaron por información recabada entre mayo y diciembre de 2021, pero fueron sistematizados para compararlos con los hallazgos posintervención reportados en este artículo. 

Tras la intervención, y para evaluar la incidencia de esta, se replicaron los procedimientos usados para recolectar información durante el diagnóstico previo referido. Así, en las cuatro escuelas, mediante una estrategia de etnografía extensiva^29^, de junio a diciembre de 2023 se realizaron entrevistas semiestructuradas con directivos, docentes y cocineras a cargo del servicio de restauración alimentaria, observación participante y análisis de bitácoras de menús cíclicos ofertados por el servicio. En cada escuela estas actividades se realizaron por un nutricionista con formación en antropología social durante dos estancias con pernocta de cinco a siete días en las localidades donde se ubican las escuelas. 

Las narrativas obtenidas mediante las entrevistas, una vez transcritas, se analizaron conforme a su función referencial, es decir, según su orientación hacia el referente o contexto mediante el uso denotativo del lenguaje para describir hechos verificables y explicar fenómenos^30^. 

Además, con base en Correas^19^, se identificaron las normas que orientan la oferta de alimentos en cada escuela mediante el análisis complementario de las conductas observadas empíricamente y del sentido que las personas entrevistadas confirieron a dichas conductas en las narrativas; es decir, cuando las personas entrevistadas explicitaban el sentido mediante enunciados que apelaban a alguna prescripción (permisión, prohibición u obligación) correspondiente a normas jurídicas (disposiciones oficiales regulatorias de la oferta alimentaria en las escuelas) o a normas comunitarias (consensos por acuerdo entre instancias de la comunidad escolar o por asamblea, no necesariamente escritos). 

Así, en las conductas observadas en los puntos de oferta de alimentos en la escuela se verificó la efectividad/inefectividad de los enunciados prescriptivos o prescripciones jurídicas y/o comunitarias referidas en las narrativas. En tales términos, el concepto *efectividad* refiere al cumplimiento de normas, es decir, cuando las personas interpeladas realizan las conductas obligatorias o se abstienen de realizar las prohibidas^19^, al tiempo que se considera la posibilidad de encontrar pluralismo normativo^19^, que es la coexistencia de sistemas normativos alternos cuya respectiva efectividad o inefectividad es verificable. 

Por su parte, cuando en las narrativas la explicación de la oferta de alimentos en la escuela se sustentó en enunciados descriptivos relativos a procedimientos instrumentales para lograr un resultado, dichos enunciados se clasificaron como reglas técnicas^19^. Es así como en esta investigación, con base en el enfoque de la *crítica jurídica latinoamericana*^19^ se asume la diferencia fundamental entre enunciados prescriptivos y enunciados descriptivos, es decir, entre normas y reglas técnicas respectivamente.

Antes y después de la intervención, la estructura y la composición de los menús ofertados se indagó por dos vías: a) revisión de bitácoras de menús ofertados y/o b) verificación y registro por observación directa. Asimismo, entre las escuelas, el ciclo de menús analizado osciló entre dos y seis semanas seguidas, con días consecutivos de lunes a viernes. Los ciclos seleccionados en la fase de diagnóstico fueron entre mayo-agosto del año 2021. Los ciclos seleccionados tras la intervención correspondieron al periodo septiembre-octubre de 2023. En ambos casos, tales ciclos se programaron conforme a la agenda de trabajo de campo del proyecto de investigación. Cabe especificar que, si bien el diagnóstico inicial recabó los datos de la oferta de alimentos durante el verano, mientras el diagnóstico posintervención los recabó durante el verano y un mes de otoño (octubre), las características analizadas relativas a la composición alimentaria de los menús, independientemente de las estaciones del año, son susceptibles de evaluación permanente conforme a las guías alimentarias nacionales mexicanas^31^ y a las disposiciones jurídicas vigentes en México en materia de preparación, distribución y expendio de alimentos en las escuelas^32^. 

Así, las características de la composición alimentaria de cada menú diario registradas fueron, en formato de lista de cotejo binaria (sí/no), las siguientes:

1. Una o más elaboraciones culinarias o productos alimentarios presentan ingredientes ultraprocesados conforme a la clasificación Nova^33^.
2. Incluye postre con azúcares añadidos.
3. Oferta exclusiva de agua como bebida.
4. Incluye todos los grupos generales de alimentos recomendados en las guías alimentarias nacionales mexicanas, que son a) frutas y verduras; b) cereales integrales o tubérculos; c) leguminosas y/o alimentos de origen animal, y d) grasas saludables^31^.

Antes y después de la intervención, estas características se procesaron como variables dicotómicas y se analizaron como frecuencias y porcentajes mediante la prueba exacta de Fisher de dos extremos, considerando cambios significativos cuando el valor de *p* fue menor a 0,05. Conviene precisar que dicha prueba es, por definición, no paramétrica y, por ende, su aplicación no busca contrastar hipótesis sobre parámetros en alguna población^34^, más bien, el propósito es valorar el efecto del azar en los cambios registrados tras la intervención.

Tales características del menú se tomaron como indicadores de cumplimiento normativo, porque se relacionan directamente con el seguimiento u omisión de disposiciones jurídicas en materia de oferta alimentaria en las escuelas mexicanas de educación básica. Además, en dos escuelas (50% del total) se identificó la existencia de kioscos escolares alternativos al servicio de restauración alimentaria, lo cual está permitido conforme a la normatividad vigente en materia de oferta de alimentos en las escuelas en México. La oferta en este tipo de establecimientos se analizó con los criterios 1 a 3, ya mencionados, para valorar las características de los menús en el servicio de restauración alimentaria.

Para sistematizar los hallazgos cuantitativos y cualitativos de antes y después de la intervención, se utilizó la propuesta analítica llamada *debilidades, amenazas, fortalezas y oportunidades* (DAFO) adaptada al diagnóstico del estado de nutrición poblacional^35^. En tales términos, se tomaron por *debilidades* aquellas características de las escuelas que producen una posición desfavorable para el cumplimiento normativo en educación alimentaria y nutricional. Como *amenazas*, las situaciones provenientes del entorno que podrían desfavorecer tal cumplimiento. En contraparte, se tomaron por *fortalezas* las capacidades especiales de las escuelas que les confieren una posición privilegiada frente al problema del cumplimiento normativo en educación alimentaria y nutricional, mientras por *oportunidades* se clasificaron las circunstancias del entorno que resultan favorables para impulsar dicho cumplimiento. 

Respecto a la intervención, a partir del diagnóstico DAFO y la disponibilidad de recursos, se priorizó diseñar e implementar una intervención basada en cuatro talleres -realizados enero de 2022 y julio de 2023- orientados a solventar las *debilidades* y optimizar las *fortalezas* (Tabla 1). Con base en un enfoque constructivista, las secuencias didácticas de los talleres priorizaron la producción grupal^36^ a partir del diálogo, la creatividad, el trabajo colaborativo y actividades lúdicas conforme a los principios de Piaget^37^ y Vygotsky^38^. En los talleres 1 y 2 se construyó gradualmente una línea de base de aprendizajes que permitió a todas las personas participantes contar con los conocimientos, actitudes y prácticas necesarios, es decir, con una zona de desarrollo próximo^38^, para culminar dichos talleres con la realización de diversas preparaciones culinarias saludables, degustaciones y experimentación en los comedores escolares. El taller 3 sirvió como plataforma para el diseño e implementación de proyectos de enseñanza-aprendizaje aplicados por el profesorado en la cotidianidad de su trabajo docente.

**Tabla 1:** Talleres para promover el cumplimiento normativo en educación alimentaria y nutricional en cada una de las escuelas participantes. Región central de México, enero 2021-julio 2023.

Número y nombre del taller	Objetivos	Dirigido a	Sesiones (n)
Evitación de alimentos con azúcares añadidos en la escuela	Identificar los riesgos nutricionales relacionados con el consumo excesivo cotidiano de azúcares añadidos. Diseñar y preparar alternativas culinarias sin azúcares añadidos.	Cocineras y comités de profesorado responsable del servicio de restauración alimentaria.	3
Diseño de menús escolares	Diseñar y preparar menús completos ajustados a las guías alimentarias para la población mexicana	Cocineras y comités de profesorado responsable del servicio de restauración alimentaria	6
Promoción integral de la salud mediante la práctica docente	Desarrollar estrategias de práctica docente basadas en la complementariedad de conocimientos científicos en salud y conocimientos del profesorado	Profesorado y dirección del plantel	6
Derechos humanos en comunidades escolares	Proponer estrategias de intervención enfocadas en la garantía y respeto del derecho a la salud, la alimentación y la educación en escuelas	Profesorado y dirección del plantel	1

Fuente: Elaboración propia. Notas: Cada sesión duró 120 minutos y el espacio entre sesiones fue de dos a cuatro semanas. Además, el taller 3 fue reforzado con la implementación de infraestructura para huertos pedagógicos escolares.

Este estudio formó parte de un proyecto nacional de investigación e incidencia, del Programa Nacional Estratégico en Salud, del Consejo Nacional de Humanidades, Ciencias y Tecnologías (Conahcyt), México. Fue evaluado con dictamen favorable por el comité de ética en investigación de la Coordinación de Proyectos Nacionales de Investigación e Incidencia en Salud Comunitaria del Conahcyt, con el registro CAR-3003. Se obtuvo el consentimiento informado de todas las personas participantes. La identidad de las escuelas participantes se resguardó al asignarles el nombre del municipio en el que se ubican. Las grabaciones y transcripciones de las entrevistas se almacenaron en un ordenador al cual solo dos miembros del equipo de investigación tuvieron acceso por contraseña. Adicionalmente, se asignó una contraseña a cada archivo de audio y de transcripción, y un código para sustituir el nombre de la persona entrevistada. Tal código se compone por dos iniciales, la primera correspondiente al municipio donde se ubica la escuela y la segunda al papel de la persona (D = dirección, P = Profesorado, C = Cocinera). Cuando en una escuela se entrevistó a dos o más personas con el mismo papel, se añadió al código un número único según el orden de participación, comenzando por el No. 1.

## Resultados

Las cuatro escuelas participantes se ubican en entornos periurbanos que, durante el siglo XX, se integraron a la expansión urbana del Valle de México y de sus alrededores. En adelante, se hará referencia con el nombre del municipio en el cual se ubican. Las escuelas de Apaxco, Estado de México, de Atitalaquia, Estado de Hidalgo, y de Xochitepec, Estado de Morelos, son de educación primaria. La de Iztapalapa, Ciudad de México, es un plantel de educación preescolar, de allí la diferencia especifica en el rango de edad que presenta el alumnado de este plantel respecto a los demás (Tabla 2).

**Tabla 2:** Perfil sociodemográfico básico de las escuelas participantes. Región central de México, 2020-2022.

Escuela	Alumnado		Profesorado		Pobreza total en el municipio donde se ubica la escuela		Vulnerabilidad en el municipio donde se ubica la escuela	
	Personas(n)	Rango de edad(años)	Personas(n)	Índice de feminidad(%)	Moderada(%)	Extrema(%)	Por carencias sociales^c^(%)	Por ingresos(%)
Apaxco	302	5-12	12	58,3	46,5	6,3	24,2	9,5
Atitalaquia	264	5-11	12	75,0	27,6	1,4	30,4	7,0
Iztapalapa	66	2-5	7	100,0	37,3	6,6	22,2	10,5
Xochitepec	140	5-13	9	88,9	52,4	8,8	20,5	7,7

Fuentes: entrevistas semiestructuradas realizadas en cada escuela en 2021 y 2022, y datos del Censo de Población y Vivienda 2020 disponibles en la web oficial gubernamental DataMEXICO^28^. ^c^Las principales carencias fueron: acceso a la seguridad social, a los servicios de salud y a la alimentación.

Además, en la Tabla 2, el índice de feminidad muestra que invariablemente es mayoritaria la presencia de profesoras como responsables a cargo de la docencia frente a grupo. Asimismo, se presentan los indicadores municipales de pobreza y vulnerabilidad (Tabla 2), a partir de los cuales es factible afirmar que en los cuatro municipios la mayoría de la población presenta vulnerabilidad -implícita en la categoría pobreza o explícita en la categoría vulnerabilidad conforme a los criterios definidos en la nota al pie- condición necesaria que justificaría la implementación del programa de desayunos escolares calientes a través de los servicio de restauración alimentaria en los términos antes definidos. 

### Diagnóstico pre-intervención

Conforme a las categorías del análisis DAFO para el logro de cumplimiento normativo en educación alimentaria y nutricional, en cada categoría se identificaron ciertos aspectos compartidos por todas las escuelas, pero también otros específicos de cada plantel. A continuación, se presentan secuencialmente los principales resultados relativos a las *debilidades*, *amenazas*, *fortalezas* y *oportunidades*. En cada subapartado, se inicia con la exposición de los resultados más frecuentes entre las escuelas y después se exponen los que ocurrieron con menor frecuencia e incluso de forma particular en algún plantel. 

#### Debilidades

En todas las escuelas se identificó desconocimiento de las guías alimentarias para la población mexicana^31^ por parte del personal operativo del servicio de restauración alimentaria (Tabla 3), el cual diseñaba los menús intuitivamente. En todos los casos, en los menús se observaron las siguientes áreas de mejora: oferta diaria de bebidas artesanales de fruta endulzadas con azúcar, postres con azúcares añadidos, proporción excesiva de cereales respecto a otros grupos de alimentos. Además, excepto la escuela de Xochitepec, estado de Morelos, en las demás, las elaboraciones calientes^25^ incluían embutidos ultraprocesados (mortadela y salchichas) de bajo costo. Más adelante, en el subapartado “Efecto de la intervención en la composición de los menús ofertados en el servicio de restauración alimentaria” se presentan los indicadores de tal oferta.

Otra debilidad, identificada en un 50% de las escuelas, fue una deficiente estrategia de control de calidad de los menús (Tabla 3). El indicador para emitir tal juicio fue la inexistencia de bitácoras de menús (o alguna alternativa análoga), las cuales son un elemento clave en la gestión del servicio de restauración alimentaria para analizar, evaluar y monitorear hábitos, gustos y preferencias, estacionalidad de materias primas, aplicación de principios de las guías alimentarias, costos, trazabilidad e identificación de puntos de control de riesgos^25^. Asimismo, permiten al personal de nuevo ingreso al servicio de restauración alimentaria reproducir menús adecuados previamente implementados^25^.

**Tabla 3:** Debilidades y amenazas para el cumplimiento normativo en favor de la educación alimentaria y nutricional en cuatro escuelas mexicanas de educación básica antes de la intervención comunitaria. Región central de México, diciembre 2021.

Escuela	Debilidades	Amenazas
Apaxco	Desconocimiento de guías alimentarias por personal a cargo del servicio de restauración alimentaria.	Venta de alimentos con alta densidad energética y escaso valor nutricional en el entorno próximo.
	Existencia de kiosco escolar.	
	La alimentación en el plantel no es parte de la educación alimentaria y nutricional.	
Atitalaquia	Desconocimiento de guías alimentarias por personal a cargo del servicio de restauración alimentaria.	El apoyo gubernamental es vertical y limita la agencia comunitaria.
	Deficiente estrategia de control de calidad de menús en el servicio de restauración alimentaria.	
	Existencia de kiosco escolar.	
	La alimentación en el plantel no es parte de la educación alimentaria y nutricional.	
	Representaciones sociales sobre los derechos humanos.	
Iztapalapa	Desconocimiento de guías alimentarias por personal a cargo del servicio de restauración alimentaria.	Sin apoyo gubernamental.
	Representaciones sociales sobre la ingesta de endulzantes calóricos.	Venta de alimentos con alta densidad energética y escaso valor nutricional en el entorno próximo.
Xochitepec	Desconocimiento de guías alimentarias por personal a cargo del servicio de restauración alimentaria.	Venta de alimentos con alta densidad energética y escaso valor nutricional en el entorno próximo.
	Deficiente estrategia de control de calidad de menús en el servicio de restauración alimentaria.	Sin apoyo de gobiernos estatal ni federal para el servicio de restauración alimentaria.
	Representaciones sociales sobre la ingesta de endulzantes calóricos.	

Fuente: Elaboración propia basada en reportes previos^5^^,^^22^^,^^23^.

La tercera debilidad, identificada en un 50% de las escuelas, fue la existencia de kioscos escolares que vendían alimentos con alta densidad energética y escaso valor nutricional (Tabla 3). En estas escuelas con kioscos, la de Apaxco, estado de México, y la de Atitalaquia, estado de Hidalgo, la cuarta debilidad consistió en inutilizar la ingesta alimenticia en el plantel como recurso didáctico para el aprendizaje de hábitos alimentarios saludables (Tabla 3). Dicha ingesta, sustentada por alimentos del kiosco escolar y del servicio de restauración alimentaria, difería de los contenidos temáticos de educación alimentaria y nutricional revisados en el aula. Así, existía un currículo oculto: en el aula se hablaba de las guías alimentarias promovidas en los libros de texto pero, en los espacios de comensalidad, alumnado y profesorado consumían mayormente alimentos densos en energía, altos en azúcares añadidos, sodio y grasas saturadas.

Otra debilidad fueron las representaciones sociales^39^ sobre la ingesta de endulzantes calóricos identificadas en un 50% de las escuelas (Tabla 3). En la escuela de Iztapalapa, Ciudad de México, y la de Xochitepec, estado de Morelos, en los menús del servicio de restauración alimentaria se consideraba necesario añadir azúcar sin refinar o parcialmente refinada a las bebidas artesanales ofertadas, elaboradas con base de pulpa de fruta y agua, bajo el supuesto de garantizar nutrientes de la fruta y a la vez asegurar la aceptación de las bebidas mediante el endulzamiento reforzado. Asimismo, en el plantel de Xochitepec se suministraban como postres dulces artesanales a base de amaranto (Amaranthus), o frutos secos, endulzados con azúcares sin refinar y melazas, bajo el supuesto de que son saludables por el menor grado de refinamiento además que al consumirlos se favorece la producción local y regional. 

En el 25% de las escuelas, específicamente la de Atitalaquia, como debilidad, se identificaron representaciones sociales sobre los derechos humanos (Tabla 3). Si bien la dirección del plantel afirmaba estar consciente de que la oferta alimentaria del kiosco escolar desfavorecía la garantía del derecho a la salud del alumnado, el argumento era que los recursos monetarios transferidos por el gobierno para financiar mobiliario y equipo escolares resultaban insuficientes y, por tanto, las comisiones de venta aportadas por el concesionario del kiosco financiarían dicho mobiliario y equipo y, así, se garantizaría el derecho a la educación, motivo primordial de la escuela.

#### Amenazas

La falta de apoyo gubernamental es una amenaza para el cumplimiento normativo en educación alimentaria y nutricional en el plantel preescolar comunitario de Iztapalapa, Ciudad de México (Tabla 3). Este tipo de escuelas comunitarias tuvieron apoyo y promoción gubernamental para garantizar la educación en lugares marginados; pero, en las últimas dos décadas, paulatinamente se les ha retirado el financiamiento, aunque se reconoce su validez oficial como centros de educación inicial. Por tanto, las familias del alumnado de dicho plantel preescolar, en reconocimiento a la calidad de los servicios, sostiene los costos de operación con cuotas mensuales.

La escuela primaria de Xochitepec, estado de Morelos, recibe financiamiento del gobierno para el gasto corriente del plantel, aunque el apoyo de los gobiernos federal y estatal es nulo para la gestión del servicio de restauración alimentaria, lo cual es igualmente una amenaza para el cumplimiento normativo en educación alimentaria y nutricional (Tabla 3). Esta escuela renunció al apoyo federal y estatal transferido por el programa de desayunos escolares calientes del Sistema Nacional para el Desarrollo Integral de la Familia (DIF), porque el tipo de alimentos suministrados difería del proyecto de educación alimentaria y nutricional promovido en la escuela. Según el profesorado, tal diferencia se daba porque el programa excluye proveedores locales de alimentos frescos e incluye alimentos envasados, alimentos transgénicos como la soya texturizada, y prohíbe la inclusión en el menú de alimentos no suministrados por el programa.

Otra amenaza, identificada en el 25% de las escuelas, es un apoyo gubernamental federal, estatal y municipal al servicio de restauración alimentaria, mediante el programa de desayunos escolares calientes del Sistema Nacional para el Desarrollo Integral de la Familia, basado en la verticalidad y la limitación de la agencia de la comunidad receptora, en este caso la de la escuela primaria de Atitalaquia, estado de Hidalgo (Tabla 3). Nadie de esta comunidad conocía el origen de los alimentos ni participaba en el diseño de los menús. Esto lo realizaba, discrecionalmente, personal ajeno a la comunidad; contratado, asignado y eventualmente rotado por el gobierno local para operar el servicio de restauración alimentaria. Ciertamente, solo el 15% de la matrícula se alimenta en el servicio de restauración alimentaria. 

Por último, en el 75% de las escuelas se ofertaban alimentos con alta densidad energética y escaso valor nutricional en el entorno inmediato exterior de la escuela (Tabla 3). Esta oferta ocurría habitualmente en los horarios de ingreso y egreso del alumnado al plantel y se daba por: 1) venta ambulante, y/o 2) venta en establecimientos de comercio al por menor. El primer tipo es más invasivo porque se dispone discrecionalmente en torno a la entrada de los planteles y/o en espacios de tránsito peatonal necesario para aproximarse al plantel o retirarse de este. 

#### Fortalezas

Una *fortaleza* del preescolar de Iztapalapa, Ciudad de México, y de la primaria de Xochitepec, Estado de Morelos (50% de las escuelas) es que siempre han desarrollado enfoques de educación activa que incluyen el aprendizaje de hábitos alimentarios por el alumnado (Tabla 4). Así, la educación alimentaria y nutricional en estas escuelas integra las dimensiones cognitiva, afectiva y psicomotriz del aprendizaje y su recurso clave es la ingesta de alimentos en el servicio de restauración alimentaria. En este espacio se construyen escenarios de comensalidad que buscan generar equidad, convivencia y solidaridad entre estudiantes. Además, lo ingerido es motivo de reflexión y análisis en el aula a partir de otras asignaturas y actividades lúdicas que apuntalan los hábitos promovidos en el servicio de restauración alimentaria. En ambas escuelas, tales estrategias didácticas han sido reportadas a detalle previamente^5^^,^^23^.

**Tabla 4:** Fortalezas y oportunidades para el cumplimiento normativo en favor de la educación alimentaria y nutricional en cuatro escuelas mexicanas de educación básica, antes de la intervención comunitaria. Región central de México, diciembre 2021.

Escuela	Fortalezas	Oportunidades
Apaxco	Tiene infraestructura para diversificar la educación alimentaria y nutricional más allá del aula.	Recibe apoyo en especies del gobierno para operar el servicio de restauración alimentaria.
	Participación comunitaria amplia en la producción culinaria del servicio de restauración alimentaria.	Gobierno local disponible para apoyar proyectos escolares de salud.
Atitalaquia	Tiene infraestructura para diversificar la educación alimentaria y nutricional más allá del aula.	El gobierno financia y asegura la operación del servicio de restauración alimentaria.
	La educación alimentaria y nutricional contempla las tres dimensiones del aprendizaje.	
Iztapalapa	Educación alimentaria y nutricional con un enfoque de educación activa orientado por la pedagogía Freinet.	Pertenece a una asamblea comunitaria con la que se intercambian saberes y espacios en torno a la agroecología.
	La alimentación en el plantel es parte de la educación alimentaria y nutricional.	
	Se prohíbe ingerir alimentos ultraprocesados en el plantel.	
	No hay kiosco escolar.	
	Es una organización de base comunitaria.	
	Personal eficiente en la gestión del servicio de restauración alimentaria.	
Xochitepec	Educación alimentaria y nutricional con un enfoque de educación activa orientado por la justicia redistributiva y el aprendizaje situado.	El gobierno local apoya en especies la gestión del servicio de restauración alimentaria.
	La alimentación en el plantel es parte de la educación alimentaria y nutricional.	
	Se prohíbe ingerir alimentos ultraprocesados en el plantel.	
	No hay kiosco escolar.	

Fuente: elaboración propia basada en reportes previos^5^^,^^22^^,^^23^.

En el plantel de Atitalaquia (25% del total), estado de Hidalgo, cuyo enfoque educativo fue caracterizado en reportes previos^4^^,^^30^, también se identificaron prácticas de educación alimentaria y nutricional que integran las tres dimensiones del aprendizaje (Tabla 4) mediante la promoción del trabajo colaborativo del estudiantado con su familia, en proyectos que incluyen preparación culinaria en casa, proyectos de investigación de la oferta alimentaria en expendios de la comunidad y la vinculación de ambos a actividades lúdicas^5^. Sin embargo, el alcance de este esfuerzo se limita porque se omite involucrar en tales prácticas la ingesta de alimentos en la escuela, tanto la del servicio de restauración alimentaria como la del kiosco escolar.

La inexistencia de kiosco escolar fue una fortaleza identificada en el preescolar de Iztapalapa y en la primaria de Xochitepec (Tabla 4). Es fortaleza porque en Latinoamérica ese tipo de establecimientos invariablemente incumple el derecho a la salud del estudiantado y prioriza el lucro de las instancias oferentes de alimentos nocivos^12^. Otra fortaleza de ambas escuelas fue la prohibición -evidenciada por el cumplimiento colaborativo de las familias del alumnado- de enviar colaciones desde casa, conformadas por alimentos tales como embutidos ultraprocesados, frituras, panecillos azucarados, pan de caja, golosinas, leche de sabores endulzada y bebidas azucaradas, entre otros. Este tipo de normas son acordadas por consenso verbal en asambleas del profesorado y familiares del alumnado, aunque su transgresión no implica sanción.

Por su parte, las escuelas primarias de Apaxco, estado de México, y Atitalaquia, estado de Hidalgo (50% del total) al momento del diagnóstico contaban con infraestructura subutilizada para diversificar la educación alimentaria y nutricional fuera del aula y dentro de la escuela, a saber: espacio para implementar un huerto pedagógico escolar y el servicio de restauración alimentaria. Esta potencialidad fue considerada como fortaleza (Tabla 4).

Respecto a la gestión del servicio de restauración alimentaria, la escuela primaria de Apaxco (25% del total) exhibió como fortaleza una amplia participación comunitaria en la producción culinaria (Tabla 4). Esto se logra mediante una agenda programada que, cada seis semanas, delega la producción culinaria y el servicio de alimentos en el servicio de restauración alimentaria, a un grupo rotativo de 4 a 6 madres de familia voluntarias, coordinadas de lunes a viernes por personal docente responsable del servicio de restauración alimentaria.

Otra fortaleza identificada en el plantel preescolar de Iztapapala fue su personal ampliamente experimentado en la gestión de servicio de restauración alimentaria (Tabla 4). La operadora única de este servicio, cuyo salario se obtiene de las colaboraciones de las familias del alumnado consumidor de alimentos, durante 20 años fue cocinera en servicios de restauración industriales, en fondas de comida casera y en servicios de cocina doméstica privados, antes de asumir, desde hace dos décadas, la operación del servicio de restauración alimentaria del preescolar. Así, con una sólida experiencia es capaz de producir y suministrar de 60 a 90 menús por jornada. Esto contrasta con la productividad más baja identificada en las otras escuelas, donde en promedio se producen alrededor de 8 a 10 menús por operadora en Apaxco, 15 a 20 menús por operadora en Atitalaquia y 35 a 45 menús por operadora en Xochitepec. 

Por último, nuevamente, sobre el plantel preescolar de Iztapalapa, Ciudad de México, resalta como fortaleza el hecho de ser una Organización de Base Comunitaria (OBC)^40^ con más de tres décadas de existencia (Tabla 4). Esta OBC está integrada por una colectividad que ha identificado los problemas que afectan su comunidad (falta de acceso a educación inicial de calidad, ser una localidad dormitorio, ambiente alimentario desfavorable, pobreza, entre otros) y en el ámbito del preescolar propone soluciones mediante un control organizativo propio, consensuado entre el personal docente, el personal operativo y las familias del alumnado.

#### Oportunidades

Mediante el programa de desayunos escolares calientes del Sistema Nacional para el Desarrollo Integral de la Familia, el 50% de las escuelas, específicamente la de Apaxco, estado de México, y la de Atitalaquia, estado de Hidalgo, recibe financiamiento gubernamental para operar el servicio de restauración alimentaria (Tabla 4). El programa articula al gobierno federal, estatal y municipal, y ofrece suministro de materias primas, capacitación y supervisión, así como equipamiento e infraestructura de espacios alimentarios^26^. En la escuela de Atitalaquia, incluye además el salario mensual de dos operadoras del servicio de restauración alimentaria asignadas al plantel. 

Por otro lado, el plantel preescolar de Iztapalapa, Ciudad de México, pertenece a una asamblea comunitaria integrada por diversos colectivos pertenecientes al barrio donde se ubica la escuela. Esta asamblea es una red de apoyo y solidaridad para resolver problemas comunes. En esta red hay instancias dedicadas a la agricultura ecológica urbana, lo cual podría vincularse a la implementación de huertos pedagógicos en el plantel o a la capacitación para su mantenimiento.

La escuela primaria de Xochitepec, estado de Morelos, tras diversos movimientos sociales de exigencia comunitaria al gobierno local previamente reportados^23^ ha conseguido recibir de dicha instancia apoyo constante en especie, principalmente, materias primas alimentarias como cereales y leguminosas.

### Hallazgos posintervención

Después de la intervención enfocada en las *debilidades* y las *fortalezas*, se actualizaron los hallazgos y se organizaron según las cuatro categorías del análisis DAFO para el logro del cumplimiento normativo en educación alimentaria y nutricional; aunque las *amenazas* y las *oportunidades* se mantuvieron constantes. Por tanto, a continuación, se presentan secuencialmente los principales resultados posintervención relativos a las *debilidades* y *fortalezas*. En cada subapartado se inicia con la exposición de los resultados con mayor frecuencia entre las escuelas y después se presentan los que ocurrieron con menor frecuencia e incluso de forma particular en algún plantel. 

#### Debilidades

Si bien la intervención introdujo en las escuelas la discusión sobre el contenido de las guías alimentarias recomendadas para la población mexicana, solo el preescolar de Iztapalapa, Ciudad de México, logró solventar las áreas de mejora atribuibles al desconocimiento de tales guías. En el 75% de planteles restantes los menús ofertados cotidianamente en el servicio de restauración alimentaria modificaron en forma parcial las áreas de mejora básicas previamente identificadas respecto a las reglas de tales guías (Tabla 5). Estos resultados diferenciales podrían deberse a los patrones de asistencia/inasistencia de las personas destinatarias de los talleres, los cuales variaron según la gestión directiva del plantel. Este aspecto se retoma a detalle en el subapartado “Efecto de la intervención en la composición de los menús ofertados en el servicio de restauración alimentaria”, relativo a cambios o permanencias en los indicadores del servicio de restauración alimentaria. 

**Tabla 5:** Debilidades para el cumplimiento normativo en favor de la educación alimentaria y nutricional en cuatro escuelas mexicanas de educación básica, después de la intervención comunitaria. Región central de México, octubre 2023.

Escuela	Debilidades
Apaxco	Conocimiento parcial de guías alimentarias por parte del personal a cargo del servicio de restauración alimentaria.
	Existencia de kiosco escolar.
Atitalaquia	Conocimiento parcial de guías alimentarias por el personal a cargo del servicio de restauración alimentaria.
	Sin control de calidad de los menús ofertados en servicio de restauración alimentaria.
	La ingesta alimentaria en el plantel no es parte de la educación alimentaria y nutricional.
	Representaciones sociales sobre derechos humanos.
Iztapalapa	Superadas respecto al diagnóstico.
Xochitepec	Conocimiento parcial de guías alimentarias por el personal a cargo del servicio de restauración alimentaria.
	Sin control de calidad de los menús ofertados en servicio de restauración alimentaria.
	Representaciones sociales sobre la ingesta de endulzantes calóricos.

Fuente: Elaboración propia basada en entrevistas y observación participante en las escuelas referidas.

El control de calidad deficiente por inexistencia de bitácoras persistió en las dos escuelas donde se identificó previamente: Xochitepec, estado de Morelos, y Atitalaquia, estado de Hidalgo (Tabla 5). En el primer caso, en visitas presenciales en y posintervención se observó que los menús son planeados verbalmente por las operadoras voluntarias del comedor y un día antes, sin dejar testimonio escrito del menú ofertado. El argumento que sustentó dicha práctica fue:

*Aquí mismo todas nos ponemos de acuerdo para decidir lo que vamos a cocinar al día siguiente, porque la verdad es que nos falta tiempo a otra hora... ¿para escribir el menú? Uy no, pues tenemos que atender diario a más de 120 niños y desde tempranito estamos aquí preparando la comida y pues de aquí nos vamos a la casa para atender a la familia, entonces ni cómo hacerle*... (AC1)

Así, la omisión de bitácoras es presentada como una regla técnica^19^ orientada a la gestión del tiempo, un recurso escaso desde el punto de vista de las operadoras del comedor, por lo que ellas priorizan producir y servir las comandas del día mientras realizan su voluntariado. Además, el testimonio revela que la planeación de los menús es apresurada y verbal, sin intervención de alguna instancia externa al grupo de cocineras.

En el segundo caso, el del plantel de Atitalaquia, el argumento fue que ya existía un menú oficial impreso publicado por el Sistema Nacional para el Desarrollo Integral de la Familia, sin embargo, en ninguna de las 36 visitas realizadas en dos años por el equipo de investigación coincidió el menú programado en tal documento y el menú realmente ofertado. Con todo, en la última visita, durante una entrevista se registró el siguiente testimonio por parte de una de las cocineras:

AC2: -*Siempre seguimos el menú del DIF* [Sistema Nacional para el Desarrollo Integral de la Familia], *o sea, está en el libro, entonces no necesitamos escribirlo, aquí servimos todo lo que dice allí...*

Persona entrevistadora: -*Sí, comprendo, aunque las veces que hemos venido vimos que no coincidía exactamente lo que ofrecieron con lo que está allí en el menú del DIF ¿verdad?*

AC2: *-Ah, este, sí, fíjese que justo cuando vinieron esas veces... no nos había llegado el dinero del municipio y había fallado la despensa.*

Además, en la escuela de Atitalaquia prevalecieron la permisión de venta de alimentos con alta densidad energética y escaso valor nutricional en el kiosco escolar y, por ende, la discordancia entre la ingesta de alimentos en el plantel y la educación alimentaria y nutricional. Además, se identificó una representación social sobre los derechos humanos proclive a justificar dicha venta, a partir de la siguiente afirmación:

...*la tienda escolar* [kiosco] *nos da recursos para poder renovar materiales, mobiliario y otras necesidades que tiene la escuela, necesarias para dar las clases, entonces, si ahorita nos quedamos sin esos recursos que nos da la tienda, no podríamos cubrir esos gastos que te digo y pues entonces no vamos a poder garantizar el derecho a la educación de los alumnos ... yo entiendo que hay un derecho a la salud, pero aquí* [en la escuela] *nos toca el derecho a la educación*. (AD1)

Como se ve, hay una interpretación que asimila y reelabora las disposiciones normativas vigentes sin considerar aspectos fundamentales como la interdependencia de los derechos humanos. Conforme a Moscovici^39^ sería una representación social que se deriva de asimilar y anclar lo nuevo (la capacitación recibida en materia de derechos humanos) a la costumbre de permitir la oferta de alimentos con alta densidad energética y escaso valor nutricional dentro de la escuela. Una conciliación de lo nuevo con lo previamente conocido mediante un proceso de asimilación y anclaje de lo primero en lo segundo^39^.

En el plantel de Apaxco prevaleció el kiosco escolar con oferta de golosinas azucaradas, bajo la justificación de que estas no exhibían sellos oficiales de advertencia nutricional; sin embargo, esto ocurrió porque dichas golosinas son compradas a granel por el comerciante que las vende en el kiosco.

Por su parte, en el plantel de Xochitepec persistieron las representaciones sociales sobre la ingesta de endulzantes calóricos incorporados en dulces tradicionales ofrecidos como postres en el menú escolar. Tal persistencia podría deberse a que dicho plantel ha sido objeto de distintos proyectos académicos durante una década y allí han confluido economistas, agrónomos, antropólogos, nutricionistas, activistas y políticos, con discursos heterogéneos respecto a la alimentación correcta. Esto podría haber generado una cacofonía nutricional^20^ que derivó en representación social, es decir, asimilar y anclar lo nuevo (la diversidad de discursos) en la persistencia del consumo de endulzantes calóricos:

*Les damos postres como las alegrías (amaranto con melaza), palanquetas (maní prensado con melaza) y dulces tradicionales, o sea, de la cultura ¿no? Ayudamos así al comercio local, a los productores, o sea, comercio justo, no son chatarra ni nada de eso, son gelatina de frutas, arroz con leche, todo artesanal y sustentable, nada de marcas de comida chatarra o industrializada*. (XP1)

Igualmente, en la escuela de Xochitepec, con el fin de amortiguar la demanda de golosinas por el alumnado al salir del plantel en expendios a 100 metros de distancia, en la puerta de este se permite a madres del alumnado vender aguas y granizados de fruta natural endulzados con azúcar. Así, la oferta previa de agua azucarada con pulpa de fruta dentro del servicio de restauración alimentaria, se trasladó al exterior -la puerta- del plantel, pero, según los testimonios, con el siguiente propósito

*Dejamos que se haga esta venta, porque, es mejor que antes coman nieve con fruta, más naturales, a que compren toda la chatarra y ultraprocesado que venden más adelante en los otros puestos y tienditas, por eso les pusimos un límite, de la puerta a 100 metros no hay chatarra, aunque sí las nieves y aguas* [artesanales y azucaradas]*, pero no es lo mismo que esas otras porquerías*. (XP3)

El punto de vista manifiesto en el testimonio estaría refiriendo una regla técnica^19^ con un propósito claro: establecer una venta anticipada en términos geográficos (justo en la puerta del plantel) para evitar compras posteriores en establecimientos que a 100 y más metros del plantel, ofrecen múltiples opciones de alimentos con alta densidad energética y escaso valor nutricional. Conceptualmente, dicha regla es una estrategia de *preemptive competition*, definida como una acción anticipada de un oferente para bloquear, desalentar o dificultad la entrada o el éxito de otro u otros oferentes competidores^41^. Así, aunque la oferta deliberada de alimentos azucarados en la puerta del plantel, promovida por la propia comunidad escolar, sería un indicador de incumplimiento normativo en educación alimentaria y nutricional, en el contexto referido de dicha acción, comprendida como regla técnica, sería una valoración desfavorable en términos de cumplimiento/incumplimiento normativo en educación alimentaria y nutricional. 

#### Fortalezas

En las escuelas de Iztapalapa, Ciudad de México, y Xochitepec, estado de Morelos, las fortalezas identificadas en el diagnóstico permanecieron durante la intervención, pero en ambos casos se incorporó la implementación y uso didáctico cotidiano del huerto escolar por el 100% del profesorado (Tabla 6). Acerca de los cambios experimentados por el plantel de Iztapalapa con la intervención, se afirmó:

*Hace unos años hubo una compañera que era muy buena y sabía mucho de huertos y con su ayuda podíamos usar el huerto para las clases, lo malo es que ninguna de nosotras aprendimos y entonces, cuando ella se fue, pues ya no nos salía el huerto lo tuvimos que dejar. Ahora, todas ya tenemos las herramientas para gestionarlo y además podemos reproducirlas, sí, sí hay un cambio importante*... (ID1)

Alternamente, en el plantel de Xochitepec se verificó lo siguiente:

*Así como ves ahorita a los niños aquí, míralos, están apuntando en sus cuadernos allí frente al huerto, pero ellos hicieron* [con sus maestros] *la composta y el mantenimiento de esas plantitas, y allí normalmente las pueden contar, clasificar y hasta escribirles poemas, luego en la cosecha se las comen en el comedor de la escuela o se las llevan a su casa para comerlas... Esto ya lo hacíamos, pero ahora que han llegado varias maestras y maestros jóvenes estuvo bien el apoyo* [mediante la intervención] *para echar a andar el huerto con más fuerza, ya aquí también las compañeras experimentadas les ayudan con ideas para las secuencias didácticas y proyectos en huerto*. (XP1)

Así, a propósito de la siembra, cuidado y cosecha de plantas comestibles se diseñaron proyectos que integraron contenidos del currículo formal, desde identificación botánica hasta aritmética. 

**Tabla 6:** Fortalezas para el cumplimiento normativo en favor de la educación alimentaria y nutricional en cuatro escuelas mexicanas de educación básica después de la intervención comunitaria. Región central de México, octubre 2023.

Escuela	Fortalezas
Apaxco	Uso didáctico del huerto para proyectos educativos por la mayoría del profesorado.
	La alimentación en el plantel se usa parcialmente con fines didácticos.
	Amplia participación comunitaria en la producción culinaria del servicio de restauración alimentaria.
Atitalaquia	Uso didáctico del huerto para proyectos educativos por una minoría del profesorado.
	La educación alimentaria y nutricional contempla las tres dimensiones de aprendizaje.
Iztapalapa	Educación alimentaria y nutricional con un enfoque de educación activa orientado por la pedagogía Freinet.
	La alimentación en el plantel es parte de la educación alimentaria y nutricional.
	Se prohíbe ingerir alimentos ultraprocesados en el plantel.
	No hay kiosco escolar.
	Personal eficiente en logística del servicio de restauración alimentaria.
	Es una organización de base comunitaria.
	Uso didáctico del huerto para proyectos educativos por todo el profesorado.
Xochitepec	Educación alimentaria y nutricional con un enfoque de educación activa orientado por la justicia redistributiva y el aprendizaje situado.
	La alimentación en el plantel es parte de la educación alimentaria y nutricional.
	Se prohíbe ingerir alimentos ultraprocesados en el plantel.
	Uso didáctico del huerto para proyectos educativos por todo el profesorado.
	No hay kiosco escolar.

Fuente: Elaboración propia basada en entrevistas y observación participante en las escuelas referidas.

Respecto a la prohibición tácita de ingerir alimentos ultraprocesados, la dirección del plantel preescolar de Iztapalapa afirmó:

*...es una labor que requiere paciencia. Pero la ventaja que tenemos es que, desde que los niños se integran al plantel por primera vez, sus papás son informados sobre cuál es nuestro proyecto educativo y que en este cuidamos también la parte de la alimentación, pues les damos de comer a los niños, pero además también es una educación de sus sentidos y hasta de su motricidad, porque también trabajamos aspectos como la masticación y cómo cocinar, con algunas frutas y verduras... Claro, hay papás, sobre todo los de primer año, que llegan a presentar alguna inconformidad pero, como te digo, con paciencia les explicamos todo las veces que sea necesario, y que también es importante que si envían comida desde la casa, pues que sea sin azúcar ni ingredientes ultraprocesados, o sea nada de galletitas, leches de sabor o embutidos de mala calidad, pan de caja, golosinas... casi siempre la gente lo comprende y nos ayuda, pero, eso, siempre con los papás de primer año hay que empezar desde el principio, picar piedra, pero terminan entendiendo y participando a veces es más difícil convencerlos a ellos que a sus niños...* (ID1)

Por su parte, en la escuela primaria de Xochitepec, el propio alumnado participa activamente en la verificación de lo que se ingiere dentro del plantel, tal como se constata en el siguiente testimonio:

...*para ponerte un ejemplo, un día unos alumnos encontraron una envoltura de galletas* [marca transnacional] *en el patio* [de la escuela], *y, ¡hubieras visto! fue un drama, toda la escuela se enteró, fueron salón por salón buscando quién había comido esas galletas y, además, por qué había tirado la envoltura, porque somos una escuela libre de plástico y basura ¿eh?...nos llevaron la envoltura, aunque nunca se supo quién fue, ya no se repitió... y es que ellos saben que comer esos productos no está bien y también insistimos a los papás en las asambleas que no los envíen a la escuela con esos productos, si es necesario mejor que no traigan nada de eso y aquí les damos de comer... por eso la mayoría de la matrícula come con nosotros*. (XP1)

Tal como se propuso previamente, en ambos testimonios subyace una prohibición tácita que resulta eficaz porque desalienta una conducta^19^, mayormente, a través del diálogo. 

En la escuela primaria de Apaxco, estado de México, el 67% del profesorado incorporó las actividades en el huerto para articular proyectos educativos integradores de contenidos de las asignaturas de ciencias y matemáticas. Al respecto, la dirección del plantel comentó:

*Varios maestros ya agarraron la onda* [comprendieron] *y se dieron cuenta de que usar el huerto no era duplicar sus actividades, porque al principio creían que era algo extra a su trabajo en aula, pero ya vieron que no es así, y ahí están, al principio eran solo dos y ahorita ya tenemos ocho maestros [de doce] que trabajan regularmente proyectos en el huerto y ahí ven temas de biología, de vida saludable y hasta ejercicios de comercialización de producto, para calcular a cuánto lo venderían según los costos y, esto, allí están las matemáticas*. (AD1)

Cabe mencionar que en dicha escuela las actividades operativas de los docentes en el huerto implican gestionar, junto con su alumnado, todo el proceso de fertilización orgánica, siembra, mantenimiento y cosecha de una fracción de parcela asignada a cada docente.

Además, también en el plantel de Apaxco, se instauró “La hora de la fruta”, un momento del día para consumir, en cada aula y con supervisión docente, fruta llevada desde casa. El objetivo de esta actividad propuesta por el profesorado fue promover el aprendizaje de hábitos de consumo y de convivencia, así como buscar la colaboración de las familias con envío de fruta como colación para cada estudiante. En los casos que no se logró tal colaboración, la escuela proporcionó la fruta al estudiante, lo cual fue factible con apoyo del gobierno local. Con todo, la persistencia del kiosco escolar con oferta de golosinas genera un currículo oculto discordante con tales estrategias innovadoras de educación alimentaria y nutricional. 

Por su parte, tras la intervención, la escuela primaria de Atitalaquia, estado de México, mostró constancia en las fortalezas identificadas en el diagnóstico, sin presentar cambios, salvo la implementación y uso cotidiano del huerto escolar para proyectos educativos por una minoría del profesorado (25%) (Tabla 6). Al respecto, un docente de dicha minoría afirmó:

*Yo me eduqué en una normal rural y conozco bien cómo la tierra nos puede servir para ver todos los temas del currículo, pero hay que educar a los maestros más jóvenes porque algunos conciben ver todo allí con los alumnos metidos en el aula y como que no quieren venir... es un desperdicio del espacio que tenemos, ya lo viste, allí, el huerto que tenemos, la ayuda se les puede dar, pero*... (ATP1)

Con todo, la implementación del huerto durante la intervención ubica este recurso didáctico como algo nuevo e incipiente en dicho plantel, así como una posibilidad para involucrar más personal docente especialmente cuando dentro de la plantilla del personal hay miembros que tienen conocimientos de gestión agrícola y un uso regular de este recurso didáctico; aunque convendría verificar el origen de la indisposición del profesorado joven referido en el testimonio (incorporado al plantel durante la fase final de la intervención) ya que, ciertamente, optó por no participar individualmente en el estudio.

### Efecto de la intervención en la composición de los menús ofertados en el servicio de restauración alimentaria

Tras la intervención, en la mitad de las escuelas se redujo significativamente la oferta de alimentos ultraprocesados en el almuerzo (Tabla 7). En todas, antes de la intervención, ese tipo de oferta estaba integrada por embutidos (salchichas o mortadela como platos fuertes) y frituras de harina. Después, prevalecieron solamente las frituras saladas como postre en los almuerzos de la escuela primaria de Apaxco, estado de México, y en la de Xochitepec, estado de Morelos (Tabla 7). 

**Tabla 7:** Proporciones de almuerzos escolares con ultraprocesados, postres con azúcares añadidos, completitud y suministro de agua, antes y después de la intervención en las escuelas participantes. Región central de México, diciembre de 2021-octubre de 2023.

Estados	Ultraprocesados					Postres azucarados					Completitud					Oferta de agua				
	Presente		Ausente		Valor de p*	Presente		Ausente		Valor de p*	Presente		Ausente		Valor de p*	Presente		Ausente		Valor de p*
	n	%	n	%		n	%	n	%		n	%	n	%		n	%	n	%	
**Apaxco**																				
Antes	2	6,7	28	93,3	0,218	14	46,7	16	53,3	0,414	14	46,6	16	53,4	0,414	0	0,0	30	100,0	<0,001
Después	5	21,7	18	78,3		8	34,8	15	65,2		13	56,5	10	43,5		22	95,7	1	4,3	
**Atitalaquia**																				
Antes	5	50,0	5	50,0	0,003	0	0,0	10	0,0	0,057	5	50,0	5	50,0	1,000	5	50,0	5	50,0	0,003
Después	0	0,0	17	100,0		6	35,3	11	64,7		9	53,0	8	47,0		17	100,0	0	0,0	
**Iztapalapa**																				
Antes	14	19,7	57	80,3	0,004	3	4,3	68	95,7	0,549	57	80,3	14	19,7	0,083	7	9,9	64	90,1	<0,001
Después	0	0,0	35	100,0		0	0,0	35	100,0		33	94,3	2	5,7		35	100,0	0	0,0	
**Xochitepec**																				
Antes	0	0,0	11	100,0	1,000	1	9,1	10	90,9	0,401	5	50,0	5	50,0	0,482	0	0,0	10	100,0	<0,001
Después	1	3,3	29	96,7		8	26,7	22	73,3		19	63,3	11	36,7		26	87,5	4	12,5	

Fuentes: Bitácoras con menús consecutivos cotejadas por registro observacional diario. Notas: El registro de 2021 se basó exclusivamente en bitácoras debido al cierre del servicio de restauración alimentaria escolar en el contexto de la pandemia por covid-19 o en entrevistas con operadoras del servicio de restauración alimentaria, en las dos escuelas (Atitalaquia y Xochitepec) donde no existían bitácoras. En el 2023 a tales estrategias se añadió la observación directa. Se muestra el número y el porcentaje de almuerzos con presencia o ausencia de la respectiva característica. *Según la Prueba Exacta de Fisher de dos extremos.

Respecto a la oferta de postres con azúcares añadidos, se incrementó significativamente en la escuela de Atitalaquia (Tabla 7), mientras las demás se mantuvieron sin cambios; sin embargo, el preescolar de Iztapalapa, Ciudad de México, no exhibió reducciones significativas porque antes y después de la intervención la oferta de tal tipo de postres fue marginal y nula, respectivamente (Tabla 7).

Por otro lado, en todas las escuelas, el cambio con mayor magnitud fue el incremento de la oferta de agua pura en los almuerzos (Tabla 7), que sustituyó todas las bebidas con azúcares añadidos tanto ultraprocesadas como artesanales, que persisten -con menor frecuencia- en algunas escuelas bajo modalidades como mezclas de fruta fresca molida diluida en agua, horchatas e infusiones azucaradas.

Por su parte, la completitud únicamente presentó cambios favorables en el plantel preescolar de Iztapalapa. Así, esta escuela fue la única que tras la intervención mostró cambios favorables en todas las variables de oferta alimentaria en el servicio de restauración alimentaria mejorables, identificadas en el diagnóstico. 

Un posible determinante de las diferencias entre escuelas en la Tabla 6, en términos del impacto de la intervención, fue la asistencia a los talleres por parte de las personas operadoras del servicio de restauración alimentaria. En el plantel preescolar de Iztapalapa, la dirección dispuso la agenda de manera tal que la operadora del comedor asistiera a todas las sesiones de los talleres, mientras en la escuela de Apaxco, las operadoras, que son madres de familia voluntarias, solo asistieron a un 50% de las sesiones, debido a que cada seis semanas fueron relevadas de sus actividades en el servicio de restauración alimentaria por un grupo nuevo de madres de familia. En ambas escuelas, la parte práctico-culinaria de los talleres se desplegó de forma experimental, elaborando y ofertando menús del día al alumnado asistente al servicio de restauración alimentaria en los días de las sesiones. 

En contraste, si bien los talleres se programaron para varias sesiones y el personal directivo de las escuelas previamente revisó, modificó y aprobó las secuencias didácticas, durante la implementación en las primarias de Xochitepec y Atitalaquia casi siempre asistieron personas diferentes a cada sesión y ninguna operadora del comedor, sino más bien familiares del alumnado convocados a recibir el taller en una sola sesión de dos horas. En ambas escuelas las operadoras no fueron involucradas en los talleres bajo el argumento de falta de tiempo por su carga laboral para producir menús en el servicio de restauración alimentaria. Esto se reforzó con la falta de planeación de dichos menús hasta horas antes del servicio cotidiano, lo cual impidió la posibilidad de articular las prácticas culinarias de los talleres en torno a esos menús. Con todo, los talleres se dieron en las cocinas y se realizaron análisis y discusiones sobre los menús ofertados el día del taller. Esto permitió cierto nivel de participación de las operadoras, cuando menos a nivel de escucha.

## Discusión

Esta investigación consistió en una intervención educativa para fomentar el cumplimiento normativo en educación alimentaria y nutricional en cuatro escuelas públicas de educación básica. La intervención, sustentada en talleres dirigidos al profesorado y personal operativo del servicio de restauración alimentaria y basada en un diagnóstico, se diseñó para incidir en áreas de mejora identificadas como *debilidades* y para apuntalar las *fortalezas* existentes. Entre las *debilidades* resaltaron: a) en el 100% de las escuelas el personal a cargo del servicio de restauración alimentaria desconocía las guías alimentarias para la población mexicana; b) en el 50%, se identificó una deficiente estrategia de control de calidad de los menús ofertados en el servicio de restauración alimentaria; c) en el 50% de las escuelas, la ingesta alimentaria en el plantel no era parte de la educación alimentaria y nutricional y era opuesta a los contenidos curriculares sobre alimentación saludable; y d) en el 50% existían representaciones sociales sobre la ingesta de endulzantes calóricos.

Tras la intervención, en los menús ofertados en el servicio de restauración alimentaria, el 50% de las escuelas redujo significativamente los ingredientes ultraprocesados y el 100% redujo significativamente la oferta de bebidas azucaradas; pero el 75% mantuvo los postres azucarados y menús incompletos en términos de combinar grupos de alimentos. En las escuelas sin todos los cambios favorables previstos, la formación del personal a cargo del servicio de restauración alimentaria fue incompleta por la asistencia inconstante a las sesiones de los talleres debido a la organización interna de cada plantel. En cambio, cuando el programa de asistencia fue completo (25% de las escuelas), se verificó la aplicación cabal de los temas de las guías alimentarias revisados en los talleres. Además, las escuelas sin control de calidad por bitácoras de menús (50%) se mantuvieron sin adoptar este recurso ni alguna alternativa análoga. Tal como se expuso, los testimonios sugieren una subestimación de este recurso de control de calidad, excepto cuando sí hubo una asistencia cabal a las sesiones de los talleres, por lo tanto, esto muestra la importancia de la capacitación del personal en la adopción de prácticas de producción alimentaria recomendables en servicios de alimentos^42^, como lo es la bitácora de menús.

Igualmente, después de la intervención, a nivel de la práctica docente, en el 100% de las escuelas se implementaron huertos pedagógicos, aunque solo en el 50% de los planteles todo el profesorado los incluyó como estrategia didáctica cotidiana para la educación alimentaria y nutricional mientras, en los planteles restantes, la incorporación fue mayoritaria en uno (67% del personal docente) y minoritaria en otro (25% del personal docente). Además, una de las dos escuelas que antes excluían la ingesta alimentaria en el plantel de la educación alimentaria y nutricional, incorporó parcialmente dicha ingesta como estrategia didáctica para el aprendizaje de hábitos saludables.

Ahora bien, sobre la asistencia inconstante del personal a cargo del servicio de restauración alimentaria a los talleres en las escuelas primarias de Xochitepec, estado de Morelos, y Atitalaquia, estado de Hidalgo, si bien la inasistencia de adultos a capacitaciones en ensayos comunitarios en educación alimentaria y nutricional se relaciona con la jornada laboral de los asistentes^43^, la rotación de personas por sesión se dio porque el personal directivo de los planteles canalizó la intervención hacia el modelo de charla informativa con complementos prácticos al mayor público posible una sola vez. Este tipo de educación alimentaria y nutricional convencional supone que, a mayor información, se dará el cambio de actitud y esta derivará en conductas saludables^44^ y pudo influir en tal asignación intempestiva de asistentes diferentes a cada sesión del taller. Así, ciertas prácticas educativas propias del modelo médico hegemónico^45^ no necesariamente fluyen unidireccionalmente hacia las comunidades, sino que a veces, por expectativas basadas en representaciones sobre el papel del personal de salud, las comunidades serían proclives a optar por -y reproducir- esas prácticas.

En las escuelas públicas de educación básica en Latinoamérica, los kioscos escolares son establecimientos invariablemente desfavorables para la educación alimentaria y nutricional^12^ y esto se verificó en el presente estudio. En México, dichos kioscos se denominan cooperativas escolares, pero este significante refiere a una tienda dentro de la escuela, gestionada por comerciantes privados que reciben la concesión de venta^15^. Ciertamente, en la década de 1930, el único presidente mexicano con orientación socialista, Lázaro Cárdenas, impulsó las cooperativas escolares en educación básica con el fin de educar a la población, desde la niñez, en la solidaridad y asociación en el esfuerzo^46^, de ahí tal denominación. Incluso, actualmente, está vigente el reglamento de cooperativas escolares, que afirma su función eminentemente educativa, orientada al desenvolvimiento social del educando, a la asimilación teórica de principios de convivencia y a la viculación del currículo al trabajo y consumo en las cooperativas^47^.

Mientras las prescripciones del reglamento orientadas a la educación son sistemáticamente omitidas^15^, se suele realiza una abstracción selectiva de fragmentos, como el artículo 47, en el que se afirma que el 40% del rendimiento económico neto de la cooperativa escolar debe destinarse a un fondo social para apoyar necesidades prioritarias del plantel^47^. Así, estas se anteponen y se obvia la oferta de alimentos con alta densidad energética y escaso valor nutritivo^48^. En este estudio se verificó tal narrativa por parte de la dirección del plantel de Atitalaquia, al presentar como regla técnica^19^, con fines económicos, la necesidad de sostener una cooperativa orientada a generar tal fondo social, incluso con venta de comida chatarra. Por ende, convendría revisar si son insuficientes los recursos financieros de programas federales como La Escuela es Nuestra, implementado desde el 2019, para dignificar los espacios educativos^49^. 

Además, la representación social identificada en la primaria de Atitalaquia -que persistió tras la intervención- según la cual se permite la existencia de un kiosco escolar oferente de alimentos nocivos porque genera recursos financieros necesarios para garantizar el derecho a la educación, aunque sea en detrimento del derecho a la salud, debe deconstruirse. Esto, por el principio de interdependencia de los derechos humanos, según el cual estos están vinculados y son indivisibles, es decir, no se pueden fragmentar unos de otros y, por ende, el ejercicio de un derecho está vinculado a que se garantice el resto de derechos^50^.

En contraste, tanto en el preescolar de Iztapalapa, Ciudad de México, como en la primaria de Xochitepec, estado de Morelos, desde hace décadas existe la convicción -por parte del personal docente y operativo- de evitar la existencia de kioscos escolares y de fortalecer el uso del servicio de restauración alimentaria escolar como espacio educativo para el aprendizaje de hábitos de convivencia y de salud. Incluso, se ha documentado cómo en ambas escuelas, con el servicio de restauración alimentaria se busca deliberadamente garantizar los derechos a la salud y a la alimentación del alumnado^23^. Ciertamente, estas escuelas desempeñan una gestión eficaz de sus recursos escasos y prescinden de representaciones sociales como las que justifican la oferta alimentaria nociva de los kioscos escolares para apoyar necesidades materiales de los planteles^48^.

Entre otras estrategias de educación alimentaria y nutricional en las escuelas de Iztapalapa y Xochitepec está la normatividad interna que, a partir de consensos con las familias del alumnado, se orienta a prohibir dentro del plantel la ingesta de alimentos con alta densidad energética y escaso valor nutricional. Son escuelas donde las familias no envían al alumnado con colaciones escolares conformadas por tales alimentos y donde ente el 60% y el 90% del alumnado es comensal del servicio de restauración alimentaria. Tal cumplimiento normativo podría interpetarse como hecho social conforme a Durkheim^51^ en la medida que al ser practicado por la mayoría es eminentemente coactivo y se impone desde fuera a los individuos, sin implicar sanciones explícitas; o bien, de acuerdo con Habermas^52^ podría afirmarse que, mediante el diálogo y acuerdos entre las personas integrantes de la comunidad escolar, se ha logrado un desarrollo moral posconvencional, de manera que los sujetos cumplen la norma por convicción apegada a principios y no por imposición.

En tales términos, la prohibición tácita de ingerir alimentos con alta densidad energética y bajo valor nutricional dentro del plantel, es eficaz porque logra desalentar el envío de dichos alimentos por las familias del alumnado. Si bien esta interpretación, basada en herramientas conceptuales de la crítica jurídica propuestas por Correas^19^, permite interpretar el fenomeno relativo al cumplimiento de dicha prescripción alimentaria en los contextos estudiados, también se puede abordar desde el ámbito de la crítica de la economía política latinoamericana. Así, conforme a Veraza^53^ -quien propone la existencia actual de una crisis alimentaria consistente en la sobreoferta de productos alimentarios nocivos- existe una serie de cinco fases por las cuales cursan los colectivos sociales para superar dicha crisis y, ambas escuelas -Iztapalapa y Xochitepec- presentarían los atributos correspondientes a la llamada *fase terminal*, la cual, el autor caracteriza de la siguiente manera:

Se conforma un movimiento social que confronta las instancias involucradas -incluido el Estado- en la sobreoferta de los alimentos nocivos, con el propósito de cerrar los establecimientos que producen y/o comercializan tales alimentos y revocar las normas -jurídicas o de facto- que lo permiten.^53^

Ciertamente, tanto el preescolar de Iztapalapa como la primaria de Xochitepec tuvieron desencuentros con las instancias del Estado, especialmente con el Sistema Nacional para el Desarrollo Integral de la Familia, respecto al tipo de alimentos que el programa de desayunos calientes suministraba, el cual incluía alimentos transgénicos y ultraprocesados, lo cual derivo en la confrontación y el retiro del apoyo alimentario tal como se ha reportado previamente^23^. 

Con todo, en la primaria de Xochitepec, tras la intervención, persistieron las representaciones sociales sobre la ingesta de azúcares añadidos a bebidas artesanales y a dulces regionales. Hallazgos similares se han reportado en escuelas mexicanas de educación básica^54^ y son un reto más allá de focalizar únicamente los alimentos ultraprocesados y con etiquetado nutricional. Así, aun en ausencia de ultraprocesados en contextos escolares, hay exposición a exceso de azúcares añadidos mediante un amplio espectro de alimentos artesanales sin etiquetar^22^ inadvertidos por representaciones sociales que focalizan el valor nutricional del alimento endulzado, mientras obvian el azúcar que se le añade^22^^,^^54^. 

Si bien el concepto de cacofonía nutricional^21^ se refiere a cómo la diversidad de discursos contradictorios respecto a consejos nutricionales proferidos por diferentes instancias se deriva en la confusion del consumidor, el concepto de representación social^39^ va más allá, porque es útil para referir y explicar el resultado de esa confusión; es decir, la asimilación y re-elaboración del conjunto de información cacofónica nueva se anclaría en el conjunto de reglas que conforman el saber-hacer alimentario previamente existente en los sujetos sociales, derivando en una representación social. 

Las representaciones sociales pueden ser resistentes al cambio, en la medida en que son reglas técnicas que forman parte del sentido común de una colectividad con cultura compartida^55^; no obstante, en el plantel preescolar de Iztapalapa, las representaciones relativas a los endulzantes calóricos no se evidenciaron en la evaluación de los menús posintervención. Un elemento diferencial, que pudo influir en este resultado favorable, fue la asistencia de las profesoras, la cocinera y la directora del plantel a todas las sesiones del total de los talleres a los cuales se las convocó. Por tanto, la construcción de un andamiaje^38^ a partir del diálogo en contextos prácticos, como fueron los talleres conformados por varias sesiones, y la aplicación-experimentación paulatina de los aprendizajes de cada sesión en la vida cotidiana escolar por las personas asistentes a los talleres, contribuyeron a lograr una praxis educativa tranformadora^3^ que trascendió las representaciones sociales sobre los endulzantes calóricos en tal plantel preescolar.

Ligado a lo anterior, en las síntesis de evidencias más recientes sobre estudios de intervención comunitaria enfocados en el aprendizaje de hábitos alimentarios en las infancias, se documenta que la ingesta de azúcares tiende a reducirse cuando se combinan estrategias educativas, jurídicas, mixtas y con intervenciones repetitivas en lapsos menores a un mes^56^. También hay más eficacia cuando se involucra a las familias y otros miembros de la comunidad escolar^57^ en vez de enfocarse solo en el alumnado. En esta investigación, en concordancia con dichos hallazgos, el plantel preescolar de Iztapalapa, donde fue consistente la asistencia de ciertos miembros adultos de la comunidad escolar a todo el programa formativo de talleres, hubo apropiación de los tópicos revisados y su aplicación comunitaria en intervenciones prácticas tales como reducir la exposición diaria del alumnado a azúcares añadidos y otros alimentos nocivos en el plantel (Tabla 7). En cambio, en las escuelas primarias, hubo rotación de asistentes en cada sesión y, por ende, la frecuencia de las intervenciones tendió a ser mensual, informativa y a reducirse al momento del taller, lo cual se reflejó en ciertos indicadores que permanecieron sin modificaciones favorables (Tabla 7).

Finalmente, el 21 de octubre de 2024, en México, el secretario de Educación comunicó en conferencia de prensa que desde marzo de 2025 será obligatorio el cumplimiento normativo de las disposiciones jurídicas vigentes en materia de preparación y oferta de alimentos en las escuelas mexicanas de todos los niveles^58^. Conviene notar que, desde hace más de una década, existen normativas obligatorias sobre la materia, las cuales, de haber sido efectivas, no hubieran devenido en la situación actual de sobreoferta de alimentos nocivos en las escuelas y en sus entornos. Entonces, la cuestión esencial sería más bien especificar cuáles serán las estrategias para garantizar la efectividad de tales disposiciones que regulan la preparación, distribución y la comercialización de alimentos en las escuelas. En ese sentido, los hallazgos de esta investigación representan casos particulares de lo posible, y podrían contribuir a visibilizar ciertas fortalezas, debilidades, oportunidades y amenazas que condicionan el cumplimiento normativo requerido para favorecer la educación alimentaria y nutricional en escuelas públicas mexicanas de educación básica.
